# A New Porous Hybrid Material Derived From Silica Fume and Alginate for Sustainable Pollutants Reduction

**DOI:** 10.3389/fchem.2018.00060

**Published:** 2018-03-19

**Authors:** Alessandra Zanoletti, Ivano Vassura, Elisa Venturini, Matteo Monai, Tiziano Montini, Stefania Federici, Annalisa Zacco, Laura Treccani, Elza Bontempi

**Affiliations:** ^1^Chemistry for Technologies Laboratory, INSTM and Department of Mechanical and Industrial Engineering, University of Brescia, Brescia, Italy; ^2^Department of Industrial Chemistry “Toso Montanari”, University of Bologna, Bologna, Italy; ^3^Interdepartmental Center for Industrial Research “Energy and Environment”, University of Bologna, Ravenna, Italy; ^4^Department of Chemical and Pharmaceutical Sciences, INSTM Trieste Research Unit and ICCOM-CNR Trieste Research Unit, Trieste, Italy; ^5^Petroceramics Spa, Kilometro Rosso Science & Technology Park, Stezzano, Italy

**Keywords:** raw materials, sustainable materials, hybrid porous materials, green chemistry, pollutants removal, embodied energy

## Abstract

In this work a new mesoporous adsorbent material obtained from a natural, high abundant raw material and a high volume industrial by-product is presented. The material is consolidated by the gelling properties of alginate and by decomposition of sodium-bicarbonate controlled porosity at low temperatures (70–80°C) at different scale lengths. The structural, thermal, and morphological characterization shows that the material is a mesoporous organic-inorganic hybrid. The material is tested as adsorbent, showing high performances. Methylene blue, used as model pollutant, can be adsorbed and removed from aqueous solutions even at a high concentration with efficiency up to 94%. By coating the material with a 100 nm thin film of titania, good photodegradation performance (more than 20%) can be imparted. Based on embodied energy and carbon footprint of its primary production, the sustainability of the new obtained material is evaluated and quantified in respect to activated carbon as well. It is shown that the new proposed material has an embodied energy lower than one order of magnitude in respect to the one of activated carbon, which represents the gold standards. The versatility of the new material is also demonstrated in terms of its design and manufacturing possibilities In addition, this material can be printed in 3D. Finally, preliminary results about its ability to capture diesel exhaust particulate matter are reported. The sample exposed to diesel contains a large amount of carbon in its surface. At the best of our knowledge, this is the first time that hybrid porous materials are proposed as a new class of sustainable materials, produced to reduce pollutants in the wastewaters and in the atmosphere.

## Introduction

Millions of tons of dyes and other organic compounds are discharge in the environment from many industries every year (Feng et al., 2017). Several of these compounds are toxic and threatening human life and the environment and can produce carcinogenic, teratogenic, and mutagenic effects.

In the last decades, many different approaches for waste water treatment and more specifically dye or contaminants removal have been proposed; they include membrane filtration, ion-exchange, coagulation, flocculation, microbiological or enzymatic decomposition, advanced oxidation, and adsorption (Feng et al., 2017). All these methods are currently applied and meet different degree of success. Among them, adsorption is considered a more feasible alternative due to its simplicity, ease of operation, ability to respond rapidly to changing conditions, insensitivity to toxicity, and high efficiency and convenience.

Today, the most commonly used adsorbent is activated carbon, which represents the state-of-the art. It has a high adsorption capacity for dyes, organic contaminants and heavy metals (Nayak and Pal, 2017). However, it is an expensive natural resource to produce and to regenerate (Tsang et al., 2007), and the use is often limited due to its higher cost, poor adsorbent regeneration capacity and other problems related to the disposal of the end-of-life sorbent (De Gisi et al., 2016; Nayak and Pal, 2017). Consequently, alternative lower-cost materials for substituting activated carbon are still a challenging issue.

A highly promising approach relies on the use of natural resources or by-products coming from various industrial processes. By-products pose different difficulties related to disposal (e.g., volume and toxicity). Their smart reuse may provide a four-fold advantage to environmental pollution like: (1) reduction of the quantity of by-products, (2) conversion of potentially toxic waste into safe materials, (3) development of low-cost, efficient adsorbents, and (4) pollution management at reasonable costs.

Literature reports several examples of declared low-cost adsorbents, developed from waste and by-products (Wong et al., 2016; Da Silva et al., 2017; Nayak and Pal, 2017). Nowadays, the research develops other “mesoporous hybrid-material” for adsorption (Li et al., 2014; Suresh et al., 2016; Tian et al., 2016; Wang et al., 2016). However, the declared sustainability of these alternative materials is often not quantified in respect to the natural resources commonly used as sorbents.

With respect to these issues, we here demonstrate a straightforward synthesis of a new porous, low cost hybrid-material, which can act as adsorbent and filter for organic compounds removal. The hybrid-material is obtained by combining sodium alginate (a naturally occurring, high abundant and inexpensive polysaccharide), with amorphous silica fume (a by-product derived from ferrosilicon or silicon metal alloy processing) (Rodella et al., 2014).

Sodium alginate is a natural polysaccharide readily extracted from various species of algae and seaweeds (Ikeda et al., 2000). Due to its fascinating properties, such as gelling capacity, film forming, emulsion stabilizing, biocompatibility, non-toxicity, and widely available, it has been broadly used for different applications (Augst et al., 2006).

Alginates were extensively adopted as adsorbent for ionic dyes (Li et al., 2017), wound-healing materials (Sikareepaisan et al., 2011), bone substitutes and drug delivery systems (Hess et al., 2016, 2017), food additives (Norajit et al., 2010), ceramic adsorbents and filters (Klein et al., 2012, 2013; Brandes et al., 2014, 2016), and so on. One of the most striking characteristics of alginate is its colloidal property, which allows the formation of insoluble gels in the presence of divalent cations, like calcium, via process described by the egg-box model (Johnson et al., 1997). Alginic acid is the only polysaccharide, which naturally contains carboxyl groups in each constituent residue (Ikeda et al., 2000).

However, the mechanical strength of the resultant material, depending on the bonded strength of the alginate molecular chains, attracted by the calcium ions, is generally low and the final obtained material can be easily broken by hand (Jia et al., 2003). This lack of desired mechanical properties, contributed to develop various strategies to overcome this problem, such as crosslinking (Rhim, 2004), blending with hydrophilic materials (Olivas and Barbosa-Canovas, 2008), and nanoreinforcement to produce nanocomposites (Lu et al., 2006).

In this context, the peculiar feature of alginate has been widely investigated for the development of organic/inorganic composites, receiving great attention in the last years, as advanced materials which combine some desirable properties of organic polymers (flexibility and elasticity) with those of inorganic solids (rigidity and chemical resistance) (Zou et al., 2008). Silica has been extensively used for synthesis of alginate/silica composites (Yang, 2016). Combining alginate with inorganic silica matrices offers several advantages in terms of chemical and mechanical stability (Pannier et al., 2014). Indeed, in recent literature (Choudhari et al., 2016), alginate–silica composites were proposed as a new class of materials with promising applications in several fields, such as biomedicine, biocatalysis, bioseparation, and biosensing.

To promote interaction between the alginate and silica, there are different approaches including surface modulation by the deposition of an intermediate layer of polycations (Coradin et al., 2001), or by using organic modified silica alkoxide (Sakai et al., 2001). However, the second route necessitates either solvents for dissolution of silicon alkoxide, silica or alginate surface modification (Pannier et al., 2014), or precipitating agents (Kurayama et al., 2010), and this deviates significantly from an environmental and economic benefit.

In this paper, we demonstrate a simple synthesis of a new porous hybrid-material (Bontempi et al., 2017 Italian Patent deposited), obtained by using sodium alginate and silica fume derived from ferrosilicon or silicon metal alloy processing (Rodella et al., 2014). In addition, some considerations about economical and environmental advantages in this use are reported and discussed. In particular, the suitability of the hybrid material as inexpensive, regenerable alternative to activated carbon for water remediation is demonstrated. Finally, preliminary results about the capability of the material to capture diesel exhaust particulate matter (PM) are reported, according to the European Commission guidelines to develop affordable, sustainable, and innovative design-driven material solution that can reduce the concentration of PM in urban areas (Materials for Clean Air—Horizon call).

## Experimental section

### Materials

Methylene blue (MB, CAS number 7220-79-3), calcium iodate (Ca(IO_3_)_2_, CAS number: 7789-80-2), sodium alginate (SA, CAS number: 9005-38-3, viscosity c = 1% water @ 25°C 5.0 – 40.0 cps), tetrakis(dimethylamido)titanium(IV) precursor (TDMAT, 99.999% purity CAS number 3275-24-9), D-(+)-Glucose (CAS number 50-99-7, ≥ 99.5% w/w), sodium carbonate Na_2_CO_3_ (CAS number 497-19-8, ≥ 99.8% w/w), sodium bicarbonate NaHCO_3_ (CAS number 14455-8, ≥ 99.8% w/w), and activated carbon (CAS number 7440-44-0) were purchased from Sigma Aldrich. To prepare samples, food grade calcium bicarbonate was bought in a local store. Silica fume was kindly provided by Metalleghe SPA, Brescia, Italy, as an industrial by-product derived from ferrosilicon and silicon metal alloy processing. The chemical composition of silica fume is reported in Rodella et al. (2014).

Double deionized water (Millipore DirectQ-5 purification system) was used for the preparation of various solutions and for Atomic Layer Deposition (ALD). Phosphoric acid (H_3_PO_4_, CAS number 015-011-00-6) was bought from Bernd Kraft.

### Synthesis of porous materials

The silica slurry was prepared using a process adapted from Brandes et al. (2014). −0.6 g SA (gel-former) were dissolved in 25 ml double deionized water (solvent) at room temperature (RT) and mixed till complete dissolution. Afterwards 1 g CI (cross-linker) was rapidly added to the SA solution under continuous stirring and a gel rapidly formed. Then 17.88 g silica fume (corresponding to 72% w/w of solid content) was added and finally 5 g of sodium bicarbonate was thoroughly mixed to the slurry.

Two ml slurry was then put in round molds and warmed on a heating plate at 70–80°C for 1 h. At this temperature the Ca(IO_3_)_2_ solubility increased and more rapid release of Ca^2+^-ions fastened the gelation process of SA and the consolidation of the hybrid materials. Simultaneously sodium bicarbonate thermally decomposed and the consequent release of CO_2_ induced pore formation. At these conditions porous disks with a thickness of 0.5 cm and a diameter of 1.5 cm were obtained.

To remove unreacted components, samples were rinsed with double deionized water and dried at ambient conditions. Whole or crushed porous disks were used for further characterization and for better comparison, some samples were not washed.

**Figure 1:**
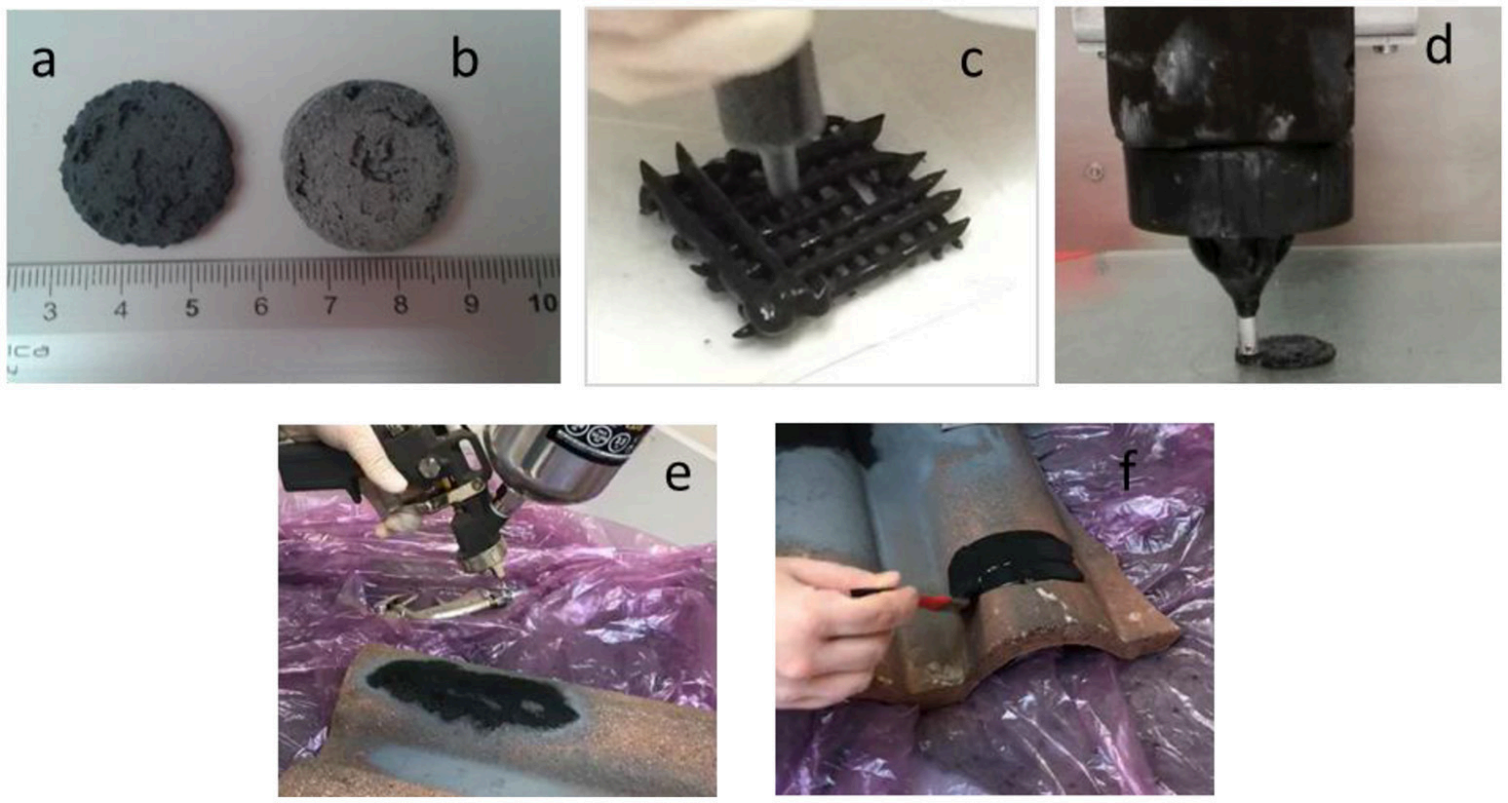
New porous hybrid material obtained by direct foaming: sample not treated **(a)**, sample treated with thermal treatment at 400°C **(b)**, extrusion **(c)**, 3D printing **(d)**, deposited by spray **(e)**, or by brush **(f)**.

The silica slurry obtained as described above and before annealing at 70–80°C has a paste-like consistence and resulted to be rather versatile and well-suited for direct foaming, extrusion, 3D printing or coatings (see Figure 1). 3D-printed structures were obtained using a self-built 3D printer (3D Maker Lab).

### Thin TIO_2_ film deposition

To improve photocatalytic activity of the hybrid material, a 100 nm thin titanium oxide film (TiO_2_) was deposited onto some samples readily after synthesis by Atomic Layer Deposition (ALD, Cambridge Nanotech Inc., Savannah system).

TiO_2_ deposition was carried out as reported in Borgese et al. (2012) using TDMAT and double deionized water as titanium and oxygen source, respectively.

To transform the amorphous as-deposited TiO_2_ film into anatase, a thermal annealing at 400°C for 3 h was performed according to Bontempi et al. (2010).

### Characterization of porous materials

Structural and chemical characterization was performed by X-Ray Diffraction (XRD), Infrared Spectroscopy (IR), porosity, nitrogen (N_2_) physisorption, Total Organic Carbon (TOC) and Thermal Gravimetric Analysis (TGA).

XRD measurements were made with X'Pert Pro diffractometer (Panalytical) equipped with the X'Celerator detector and Cu anode (CuKalpha 1.5406 A) operating at 40 KV and 40 mA. The pattern was collected between 5 and 70° (in 2θ).

IR measurements were taken using the Equinox 55 (Bruker) FT-IR spectrometer. Each spectrum was recorded in triplicates from 4,000 to 400 cm^−1^ with a mean of 128 scan per sample and a resolution of 4 cm^−1^. The spectra were baseline corrected using OPUS software, version 5.0.

UV-VIS measurements were done using QE65000 spectrophotometer (Ocean Optics).

A LEO EVO 40 scanning electron microscope (SEM) (Zeiss) was used to perform a morphological characterization of the porous samples. The energy dispersive X-ray spectroscopy (EDXS) microprobe (Link Pentafet Oxford mod 7060) coupled to the SEM allowed to investigate the elemental composition of materials.

Nitrogen (N_2_) physisorption (Micromeritics ASAP 2020 analyzer) measurements at the liquid nitrogen temperature were used to investigate the textural properties of the materials. Prior measurements 500 mg of each sample were degassed at 100°C overnight.

TGA was carried out under an inert atmosphere (N_2_, 90 ml/min) using TGA/SDTA 851e analyzer (Mettler Toledo). Before analysis, samples were crushed into a fine powder in an agate mortar and about 10 mg of sample were used for measurements. For moisture content determination samples placed in alumina crucibles were heated to 105°C kept steady for 6 min, then increased to 950°C at 40°C/min, and mantained 5 minutes longer.

TOC content was set with a TOC analyzer (mod. SSM 5000A, Shimadzu) and calculated as the difference between Total Carbon (TC) and Inorganic Carbon (IC). Both TC and IC were set after the oxidation to CO_2_ using a detector working in the IR range. TC oxidation was due to the temperature (900°C) as well as to the catalyst (Pt+Co), while for IC determination, the sample was acidified at 200°C with H_3_PO_4_ 42.5%, which reacted with IC to form CO_2_. TC and IC were measured by the external standard method, using different amounts of standard samples respectively of D-(+)-Glucose and Na_2_CO_3_, as indicated by instrument user's manual.

### Adsorption of methylene blue

The organic dye methylene blue (MB) was used as a model for evaluating the adsorption behavior and capacity of the material.

Calibration curves were obtained by using standards with concentrations between 1 and 10 mg/l.

Tests were carried out by soaking the samples in MB solutions overnight to reach equilibrium conditions. Each measurement was carried out under identical experimental conditions using rinsed samples as described in the synthesis section. The samples were inserted in MB solutions (200 mg/l) with a solid/liquid concentration of 40 g/l. Samples were kept at RT in dark and constantly stirred at 300 rpm. The stability of MB was evaluated in preliminary studies, which demonstrated that at these condition MB is stable and it does not undergo any significant self-photodegradation under UV irradiation. The concentrations of MB in aqueous solutions before and after adsorption were evaluated by an UV-VIS spectrophotometer at fixed wavelength (663 nm).

The percentage of MB adsorbed by the disks was calculated by Equation (1):

(1) $$\begin{array}{l} R=\left(C_{0}-C_{t}\right)/C_{0}{\;}^{*}100 \end{array}$$

where *C*_0_ and *C*_*t*_ (mg/l) are the concentrations of MB at time zero and after a determined time *t*, respectively.

The amount of adsorption at equilibrium, *q*_*e*_ (mg/g), was calculated by Equation (2):

(2) $$\begin{array}{l} q_{e}=\left(C_{0}-C_{e}\right)V/W \end{array}$$

where *C*_0_ and *C*_*e*_ (mg/l) are the concentrations of MB at initial and equilibrium, respectively. *V* is the volume of the solution (in l), and *W* is the mass of the samples (in g).

Photodegradation experiments were carried out using samples soaked overnight in MB solutions of 200 mg/l. The disks were continuously stirred, while being irradiated with UV light (Philips UV lamp) with emission wavelength between 340 and 410 nm and with a maximum at 365 nm. The variation of degradation was monitored at different time intervals (every hour) for a total period of 6 h. Tests were carried out in triplicates in 9 different independent photodegradation experiments.

MB is stable under UV, in fact it was verified that it did not undergo any significant self-photodegradation under UV irradiation under the conditions utilized in these experiments.

MB adsorption efficiency of the material was compared with the activated carbon powder, used as reference. Rinsed porous samples were turned in powder form with an agate mortar. Two solutions containing 500 mg/l of MB were mixed with a 2.5 g/l sample or activated carbon for 6 h. The MB concentration was set to 500 mg/l which corresponds to the maximal adsorption of activated carbon. This was determined in some preliminary tests.

### Diesel exhaust PM capture

The porous material was placed at about 15 cm distance from a diesel exhaust emission source for 15 min. This test was realized to evaluate the possibility to use the porous material to capture ultra-fine particles (Chow et al., 2006).

## Results and discussion

### Material characterization

TC, IC, and TOC of the material before and after rinsing, and those of the raw materials used for sample fabrication are reported in Table 1.

**Table 1:** Data about TC, IC, and TOC of the obtained materials (sample as well and after washing).

**Sample**	**TC (mean)**	**TC s.d. (*n* = 3)**	**IC (mean)**	**IC s.d. (*n* = 3)**	**TOC**	**TOC s.d**.
Sodium alginate	26.1	0.8	<0.1			
Silica fume	1.40	0.01	<0.1			
Washed porous material	2.68	0.06	1.0	0.1	1.7	0.2
Porous material	7.4	0.2	2.3	0.1	5.1	0.3

*Data are reported in percentage. The TOC content can be calculated as the difference between Total Carbon (TC) and Inorganic Carbon (IC)*.

Silica fume turns out to be the most abundant material used for sample synthesis. It contains low amount of organic carbon (<1.4%). As expected, the amount of TOC in SA is very high (about 26%). After synthesis, the material contains about 5.1% TOC, and 1.7% after rinsing. This value, which is lower in respect to the synthesized material, is significantly higher than the one of silica fume.

**Figure 2:**
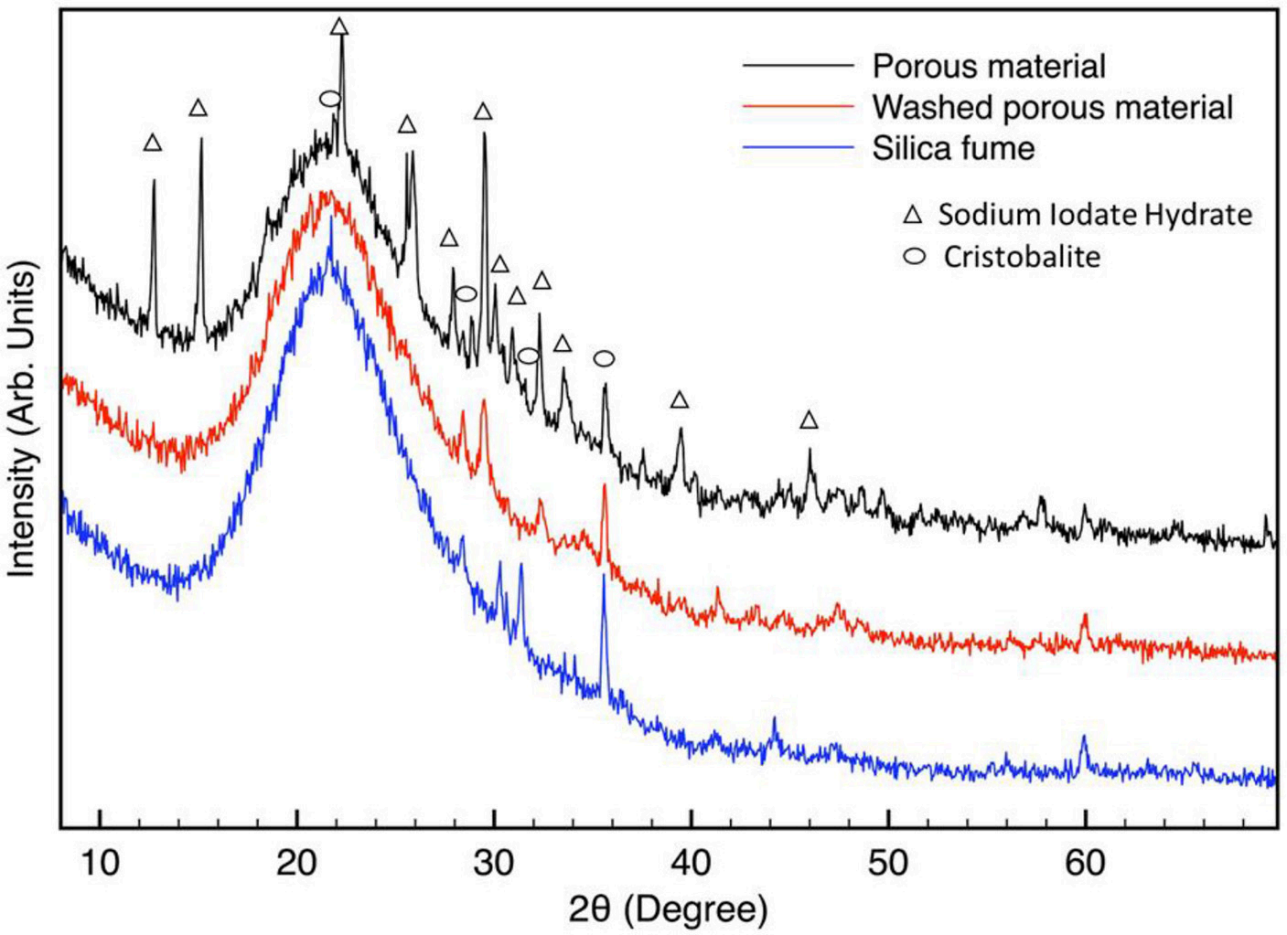
XRD spectra of synthesized porous material before and after washing. XRD pattern of silica is also reported. Sodium iodate hydrate (NaIO_3_·H_2_O, PDF CARD *n*° 321096) and Cristobalite (SiO_2_, PDF CARD *n*° 751544 and 010438).

XRD spectra are shown in Figure 2. The as-obtained sample (before washing) shows several diffraction peaks, which can be attributed to a sodium iodate hydrate phase. This phase is probably formed by the dissolution of sodium ions from alginate and the presence in the solution of iodate ions from calcium iodate used as a cross-linker. A large halo in the range of 15–30° (in 2θ) can be attributed to the presence of amorphous materials. The XRD pattern of a washed porous sample is very similar to the one reported for as-obtained sample with the absence of sodium iodate hydrate which is water-soluble and can be dissolved by rinsing. The XRD pattern of silica fume is also reported in Figure 2.

The peaks at about 30.3° and 31.4° maybe due to the presence of Cristobalite phases. Also in the washed porous sample, some peaks can be attributed to crystalline silicon oxide phase (Chen et al., 2016). Cristobalite peaks are poorly evident in the XRD pattern of not rinsed samples because of the predominance of sodium iodate hydrate phase.

The main XRD Calcite (CaCO_3_) peak falls at about 29.5°, then its presence cannot be excluded in XRD patterns of porous materials. From XRD spectra it is possible to conclude that the obtained porous material is essentially amorphous, indeed, the low temperatures used during synthesis are not sufficient to promote materials crystallization, but only consolidation. However, from data analysis it results that the amorphous halo (from about 15° to 30° in 2θ) of silica fume has an higher integrated intensity in respect to that of porous material. This may be attributed to a difference in the structural factors of silica fume and the porous materials.

**Figure 3:**
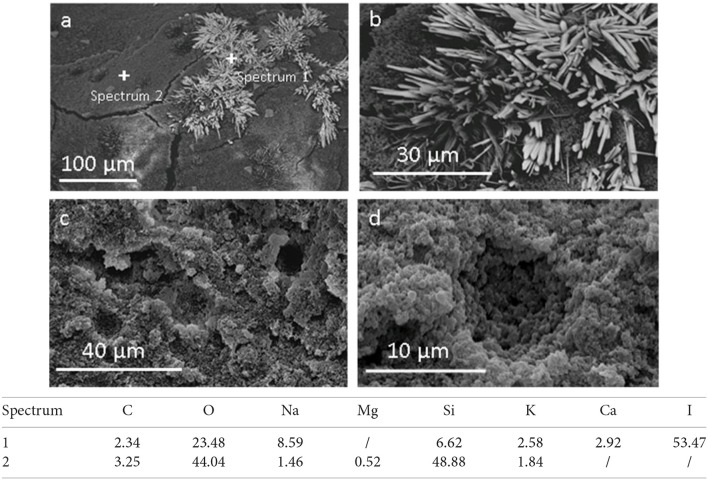
SEM analysis of porous material before **(a,b)** and after washing **(c,d)**, and semi-quantitative chemical analysis of selected points (Data are reported in weight %).

SEM images of porous samples before and after rinsing are shown in Figure 3. It is evident that the structure of the obtained materials is porous, similar to a sponge with micro and macro pores, that seem to be interconnected in between them. This is probably due to sodium bicarbonate decomposition.

Figure 3a shows the presence of some needle-like structures (details in Figure 3b) in the porous material before washing. A comparative EDXS analysis (Figure 3) reveals that these needles are mainly formed by iodine. This suggests that they are sodium iodine crystals already identified in XRD spectra. After the washing of the material the needles disappeared, as evident in the Figures 3c,d, and in accordance with XRD results. On the contrary, EDXS analysis shows that the porous matrix is characterized mainly by the presence of oxygen and silicon.

**Figure 4:**
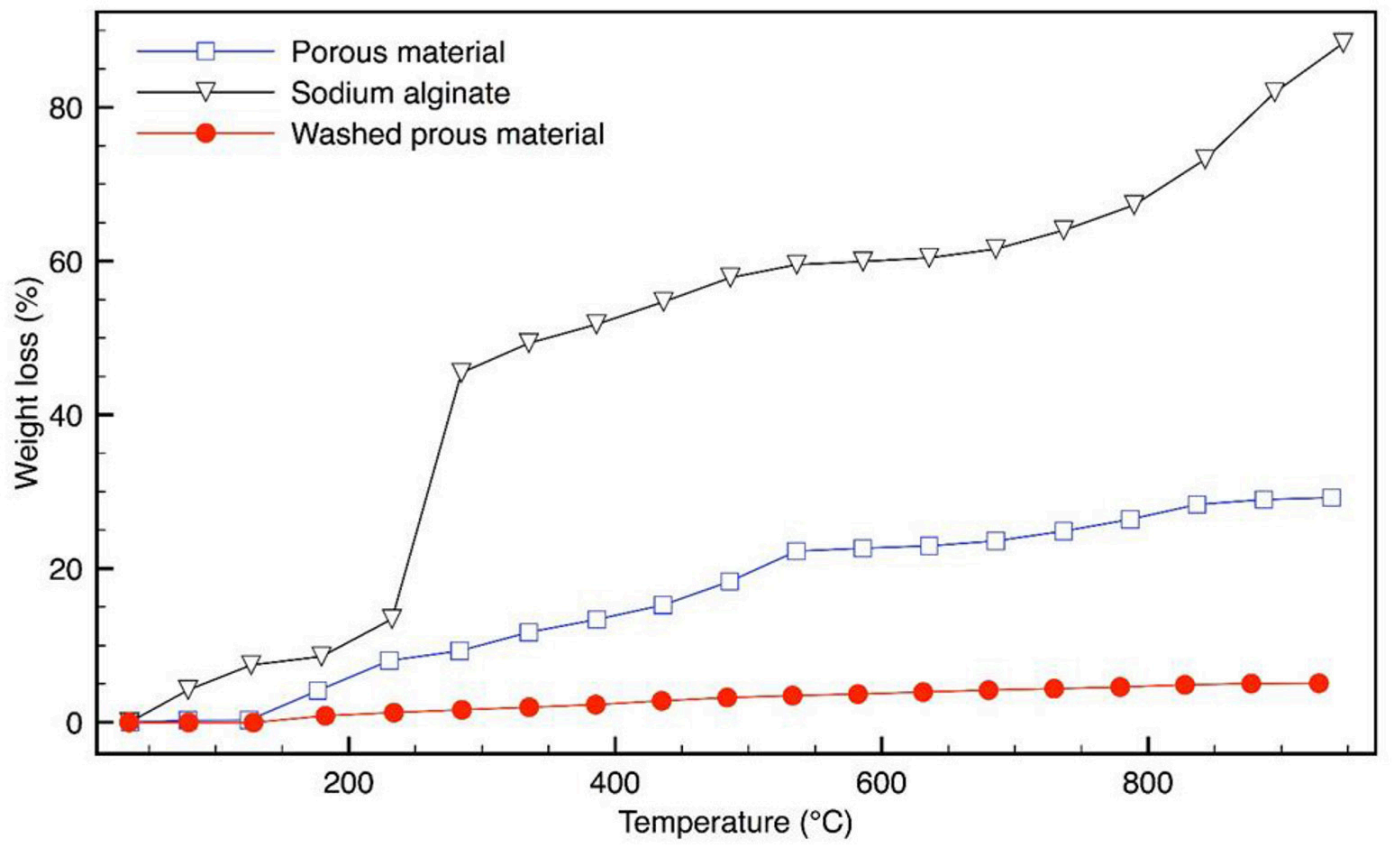
TGA made on pure SA and the obtained porous materials after and before washing.

TGA analyses are reported in Figure 4. SA shows a quick weight loss in the temperature range of 200–270°C, which can be associated with its decomposition. The weight loss of the material, reported in Figure 4, is in accordance with (Lencina et al., 2013). The mass loss, up to about 150°C can be attributed to the water molecules loss and the observed mass loss from about 150 to 260°C may be due to the residual SA decomposition and to the condensation of the IO_3_ group into I_2_O_5_ (Liu et al., 2008). The decomposition in the range of about 300–700°C can be associated to oxygen and iodine loss, in the sodium iodate hydrate phase (Girase, 2013).

The washed sample (with removal of iodate salt) shows a different thermal behavior: the mass loss up to 900°C is quite low (about 5%), indicating a wide material stability.

It is evident that the thermal stability of the obtained porous material results highly enhanced in respect to the starting pure alginate, suggesting the formation of a stable and resistant organic-inorganic network.

**Figure 5:**
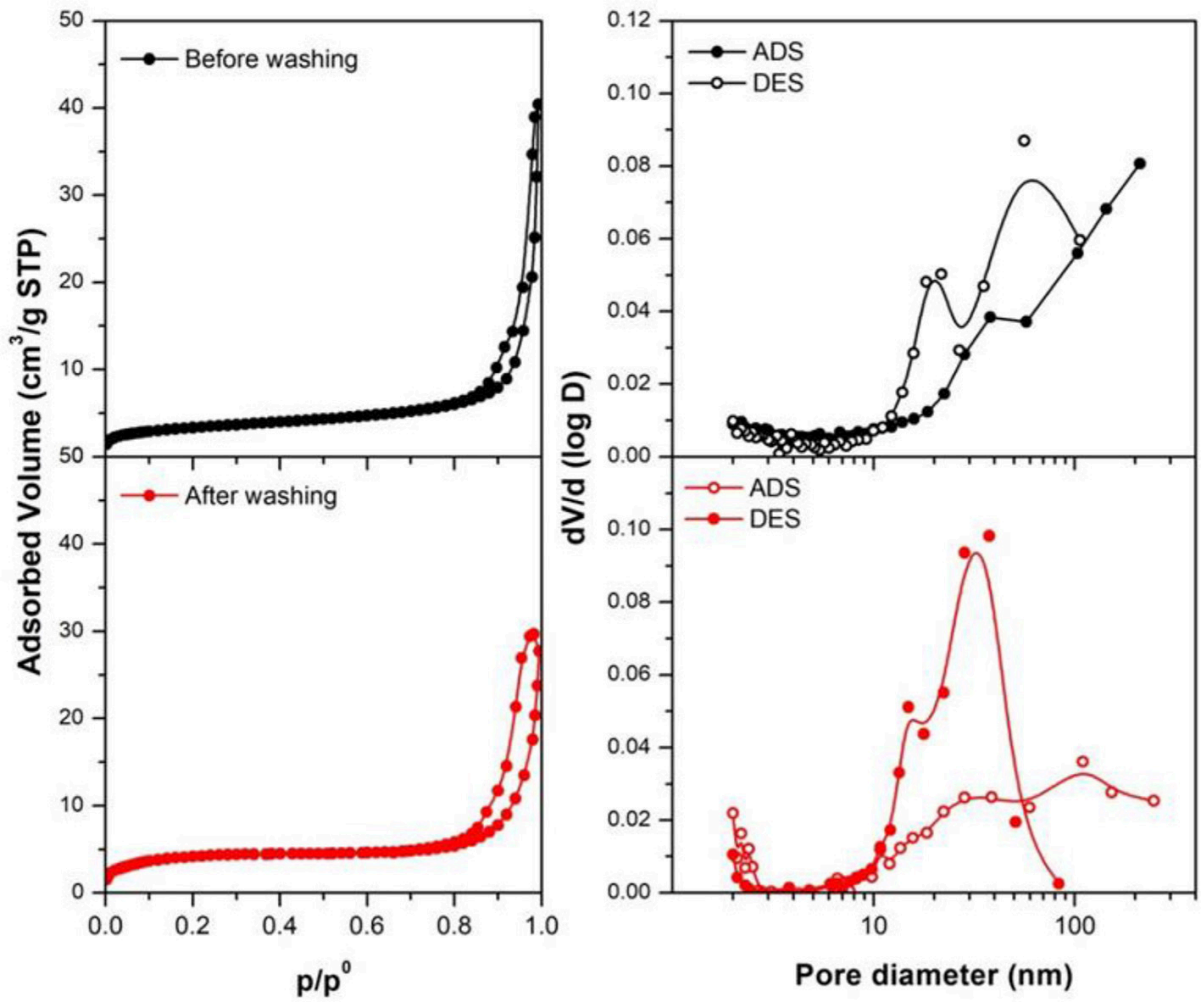
N_2_ physisorption isotherms and pore size distributions (calculated following the BJH method) for the obtained samples (before and after the washing). Measures were made in adsorption (ADS) and desorption (DES), to evaluate the pores dimensions.

The textural properties of the synthesized materials (before and after washing) analyzed by N_2_ physisorption are summarized in Table 2, while Figure 5 presents N_2_ physisorption isotherms and the correspondent pore size distributions (obtained applying the BJH method). Following IUPAC recommendations (Sing et al., 1985), all the samples present type IV isotherms, typical of mesoporous materials. The BJH pore size distribution highlight that the materials contain both mesopores and macropores. The presence of macropores is also suggested by the increase of adsorbed volume at high p/p_0_ values. Notably, the contribution of mesopores increases after washing of the samples. The specific surface areas and the cumulative pore volume of all the materials are low (10–12 m^2^/g). In agreement with this, large mesopores and macropores are observed in the pore size distribution profiles. Notably, pore size distributions calculated from the desorption branch of the isotherms show relative maxima at lower values with respect to those calculated from the correspondent adsorption branch. This indicates that ink-bottle shaped pores are present, as also suggested by the hysteresis loops observed in the N_2_ physisorption isotherms.

**Table 2:** Textural parameters of the investigated samples.

**Sample**	**S^a^_BET_ (m^2^ g^−1^)**	**d^b^_ADS_ (nm)**	**d^c^_DES_ (nm)**	**CPV^d^ (cm^3^ g^−1^)**
Porous material	10.5	39; >150	20; 60	0.060
Washed porous material	11.6	31; 110	15; 32	0.044

a *Specific surface area calculated following the BET method*.

b *Relative maxima of the pore size distribution calculated applying the BJH method to the adsorption branch*.

c *Relative maxima of the pore size distribution calculated applying the BJH method to the desorption branch*.

d *Cumulative pore volume*.

**Figure 6:**
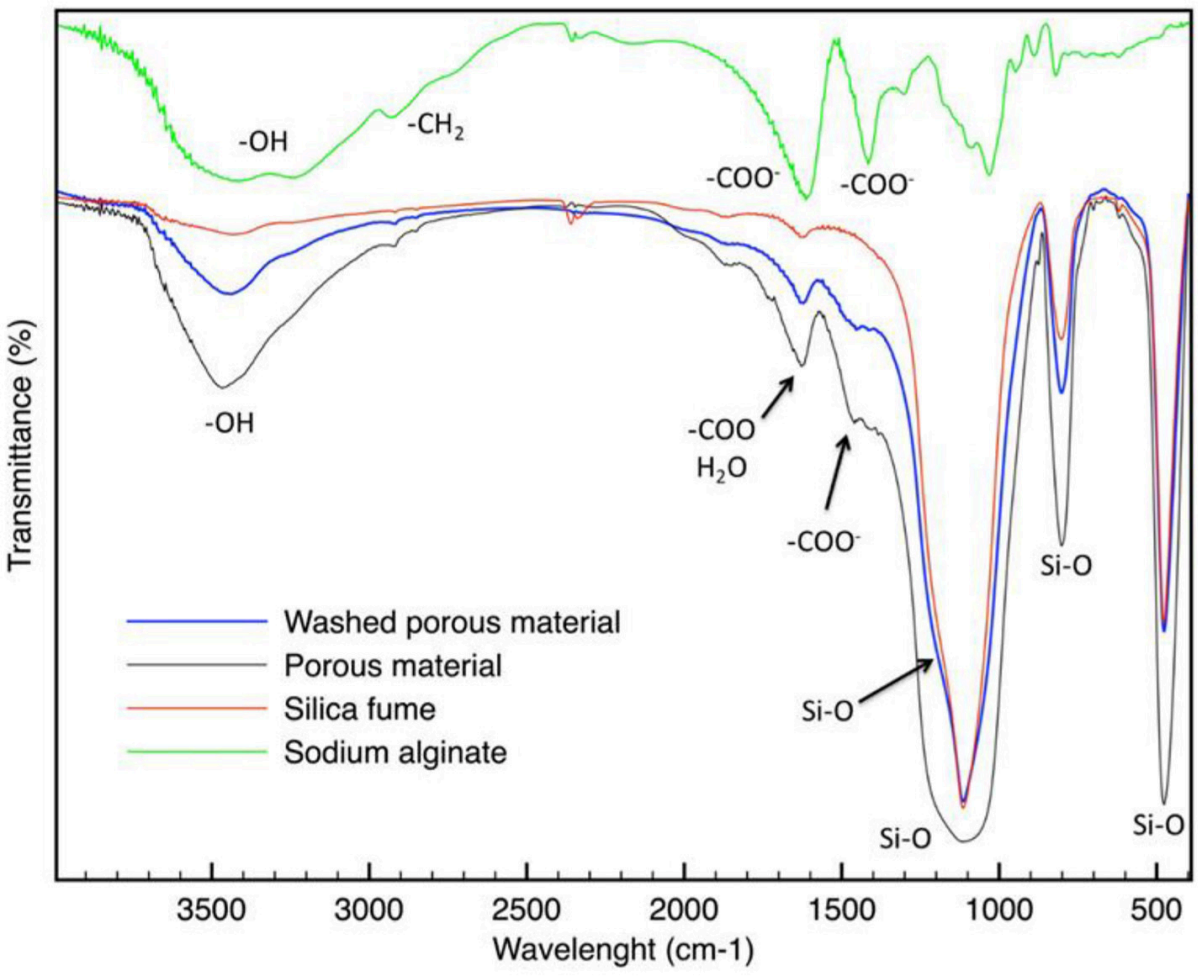
FT-IR spectrum of SA, silica fume, and the material before and after washing.

The FT-IR spectrum of SA (reported in Figure 6) shows characteristic bands observed for alginate (Pannier et al., 2014). The broad peak at about 3,400 cm^−1^ corresponds to the OH stretching vibrations of hydrogen-bonded OH groups. Other characteristic peaks of sodium alginate can be found at about 1,610 and 1,420 cm^−1^. They are respectively due to the asymmetric and symmetric stretching peaks of carboxylate (–COO^−^). The broad adsorption band in the region of 1,200–1,000 cm^−1^ is attributed to the vibrational modes of the carbohydrate rings.

In Figure 6, the FT-IR spectrum of silica fume is also reported. The intense band in the region between 1,250 and 1,100 cm^−1^ can be attributed to the Si–O–Si bonds (symmetric and asymmetric stretching). The bands at about 470 and 800 cm^−1^ are absorption bands of Si–O vibrations. A large band between about 3,300 and 3,700 cm^−1^ is also characteristic of silica: generally, the peaks below 3,500 cm^−1^ are caused to the stretching vibrations of free molecule water or water adsorbed on silica surface; the peaks above 3,500 cm^−1^ are due to the –OH stretching in silanol (Aragao and Messaddeq, 2008). Peaks of isolated silanols are associated to the band in the highest wavenumber 3,750–3,680 cm^−1^, while peaks of mono-hydrogen-bonded (3,600–3,400 cm^−1^) are located at lower wavenumber regions (Hu et al., 2016). The proximity of these peaks usually results, in highly overlapped spectra. The band at about 1,625 cm^−1^ is attributed to the bending vibration of adsorbed water molecule (Lei et al., 2016).

The FT-IR spectrum of the materials before and after washing (reported in Figure 6) are quite like silica fume. Indeed, for example, the strong adsorption bands at 470, 800, 1,100, and 1,250 cm^−1^ are attributed to the fundamental Si–O vibrations. They do not differ in the various silica modifications (Bergna, 1994).

The broad peak at about 3,400 cm^−1^ can be attributed to OH stretching vibrations of hydrogen-bonded OH groups. Because this can be related not only to the silanol groups, but also to the adsorbed water, it is very difficult to speculate about the presence of silanol groups on obtained material. However, it is interesting to highlight that around 3,200 cm^−1^ a band that can be associated to poly-hydrogen-bonded silanols appears (Hu et al., 2016).

In addition, it is interesting to note that the band between 965 and 975 cm^−1^ characteristics of calcium silicate hydrate does not appear (Yu et al., 1999), indicating that there are no products like those obtained in the cement hydration.

This evidence leads us to suppose that Ca^2+^ ions formed ion-exchange with some silanol groups on silica fume, as already reported in literature (Lei et al., 2016).

A new adsorption band, in respect to the silica fume, can be found between 1,410 and 1,450 cm^−1^. It may be attributed to the carboxylate groups (Pannier et al., 2014), deriving from alginate. This peak was observed to shift to a higher wavelength, in respect to the alginate, which could result by interaction with the silica surface. This observed peak demonstrates the successful synthesis of the hybrid material. Also the peak at 1,610 cm^−1^, corresponding to –COO^−^ asymmetric stretching is present in the FT-IR spectra. However, it corresponds also to the adsorbed water molecules. Therefore, a single attribution is not possible.

Sodium alginate can be dissolved in water at room temperature by mechanically stirring. At the addition of a divalent metallic salt solution into the sodium alginate solution, the gelation reaction takes place immediately. The proposed ion chelation mechanism involves the interaction between some polysaccharide chains and divalent ions (calcium in the present case) with the formation of a junction, as described in the so-called egg-box mode (Morris et al., 1978). This leads to the formation of the hydrogel.

A different mechanism occurs when silica fume is added to the solution. On the silica fume particles surface there are many silanol groups, which can be deprotonated (Greenberg, 1956):

$$\begin{array}{l} \text{Si}-\left[\text{OH}\right]⇄\text{Si}-{\text{O}}^{-}+{\text{H}}^{+} \end{array}$$

Ca^2+^ ions, derived from a salt, can react with the deprotonated silanol group:

$$\begin{array}{l} \text{Si}-{\text{O}}^{-}+{\text{H}}^{+}+\text{C}{\text{a}}^{2+}⇄\text{Si}-\text{O}-\text{C}{\text{a}}^{+}+{\text{H}}^{+} \end{array}$$

Then the modified silica fume by the presence of Ca^2+^ ions can adsorb different types of anions (Lei et al., 2016). By the combination of negatively charged alginate groups with the positively charged obtained silica surface, it is possible to obtain a stable material. Indeed, the modified surface of SiO_2_ can interact with the –[COO]– and –[O]– groups of sodium alginate to promote the formation of new bonds. These new interactions could decrease the additional active sites for the binding of entering water molecules and promote the final solid precipitation (Yang, 2016). This assumption agrees with the results obtained by FT-IR.

**Scheme 1:**
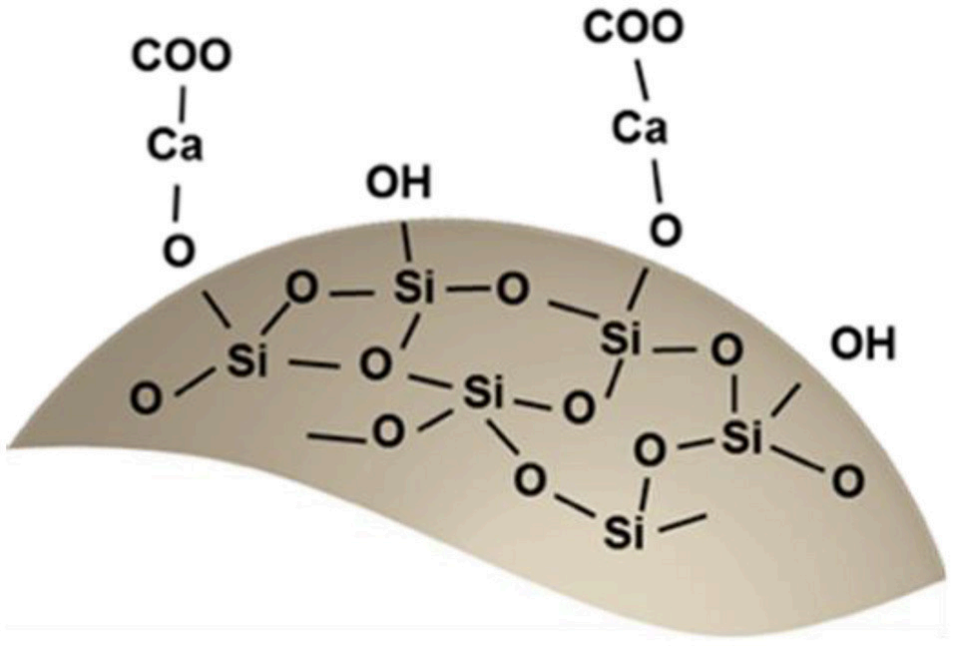
Representation of the suggested chemistry of the obtained hybrid material.

Based on these results, a schematic representation of the suggested chemistry of the obtained hybrid material is illustrated in Scheme 1.

### Adsorption and photocatalytic properties

For the MB adsorption experiments, three different typologies of mesoporous hybrid materials were tested: porous material (sample 1), porous material annealed at 400°C (sample 2) and porous material covered by TiO_2_ annealed at 400°C (for enabling anatase crystallization, sample 3). All samples were rinsed after synthesis and ALD treatment.

In Table 3, the results of MB adsorption are reported, by showing the removal efficiency, R, calculated according to Equation (2).

**Table 3:** Percentage removal efficiency (R%) of MB.

**R (%)**	
**Sample**	**Mean** ± **s.d**.
1	95.4 ± 2.5
2	97.7 ± 0.9
3	94.1 ± 0.1

*Sample 1, 2, and 3 are porous material, porous material treated at 400°C and porous material with TiO_2_, respectively*.

Results reported in Table 3 show that all materials have high and comparable adsorption properties. Apparently, it seems that the thermal annealing at 400°C and the presence of a thin layer of anatase does not have a significant influence.

The photocatalytic activity of the sample was assessed using a rinsed sample (sample 1), a sample annealed at 400°C (sample 2) and a titania coated sample annealed at 400°C (sample 3). After the overnight materials adsorption, the residual MB concentration was in the order of 4–12 mg/L.

**Figure 7:**
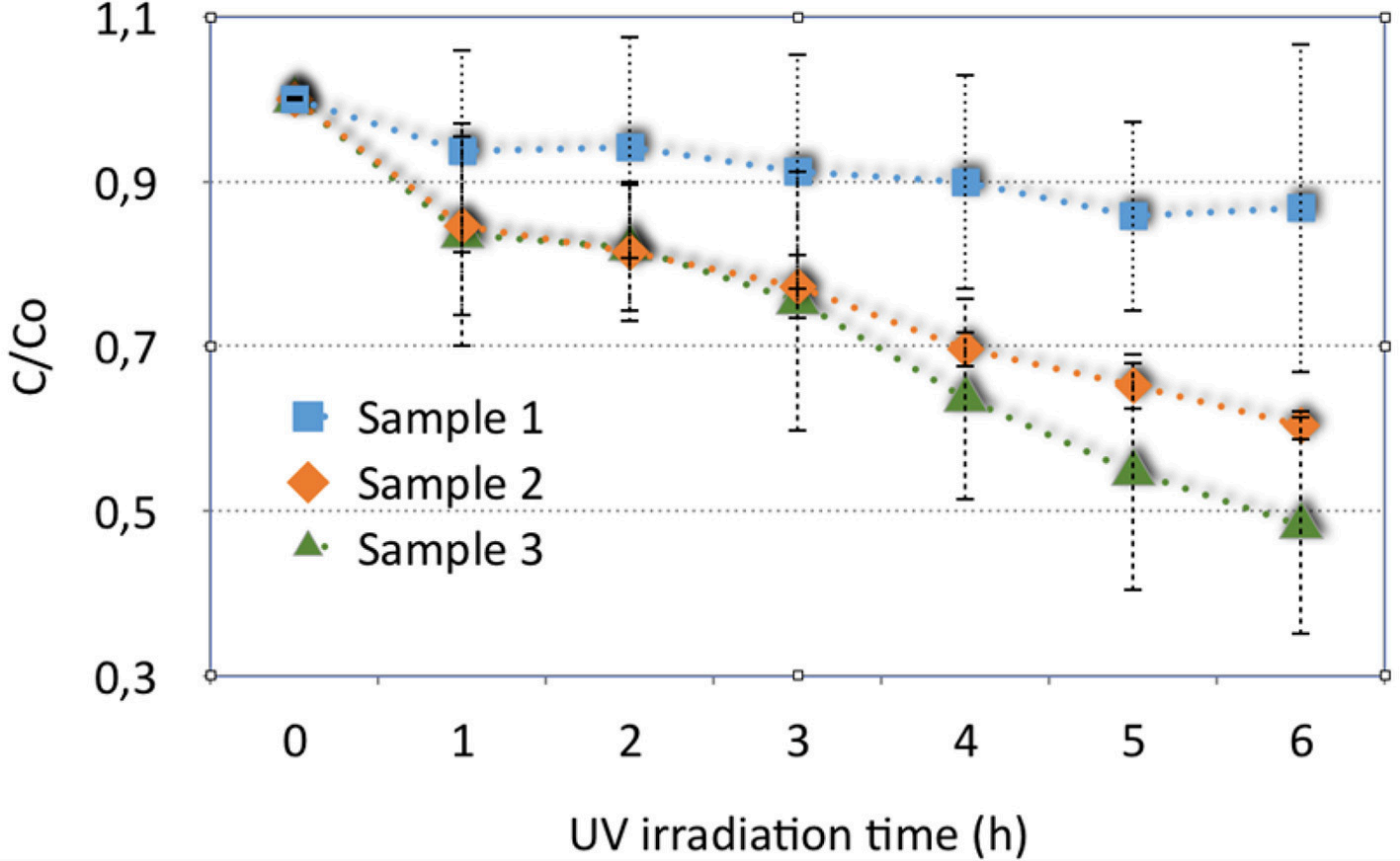
Results of photocatalytic activity of washed hybrid porous sample (sample 1), sample annealed at 400°C (sample 2) and a corresponding sample, covered by titania (sample 3).

The results of photocatalytic activity of these three samples, considering the mean of the obtained data, are shown in Figure 7, with corresponding standard deviations, reported as errors bars. Despite the variability in the results, the materials show different behaviors in Figure 7. Rinsed samples (sample 1) can degrade the solution of MB with an efficiency of about 20%.

The efficiency increases considering the same material annealed at 400°C (sample 2) and for the material covered with titania and treated at 400°C for anatase crystallization (sample 3): samples 2 and 3 show a mean percentage of degradation of about 40 and 52%, respectively.

As expected, the titania coating contributes to increase the efficiency in MB photodegradation, even if only a thin layer of TiO_2_ is deposited onto the disks. However, it is evident that the titania deposition onto the porous material disks increases the embodied energy (EE) and carbon footprint (CF) of the final obtained material. Then this possibility must be properly accounted.

### Diesel exhaust PM capture

**Figure 8:**
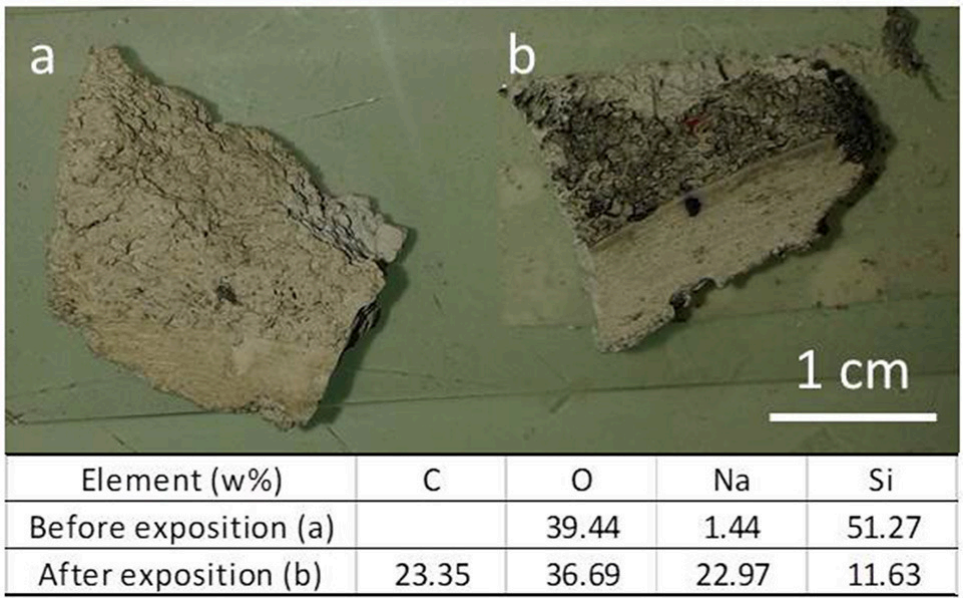
New porous material, before **(a)** and 15 min after **(b)** the exposition to exhaust fume of a diesel car.

Figure 8 reports the picture of the new porous material, before and 15 min after the exposition to exhaust fume of a diesel car. It is evident that the material porosity results very effective in capturing particles.

EDXS analysis (reported in Figure 8), made on the sample before and after exposition, revealed that the sample exposed to diesel contains a large amount (more than 23% in weight) of carbon in its surface. Indeed, diesel exhaust particles contain a carbon core generally coated by polyaromatic hydrocarbons, quinones, and metals (Williams, 2011).

Dedicated analysis and tests are in progress to better evaluate the PM trapping capability of the new material.

The results about pores shape and dimensions make it very promising for capturing ultra-fine PM (< 1 micron).

The possibility to apply it as a coating, by spray or by brush, makes it suitable to be used, for example, to cover external building surfaces.

### Material sustainability

Figure 1 shows different processes of the production of the new proposed porous material: by direct foaming (Figures 1a,b), extraction (Figure 1c) and 3D printing (Figure 1d). The new porous material can be also applied as a coating, by spray (Figure 1e) of by a brush (Figure 1f). This production flexibility can be exploited to produce new adsorbent typologies for industrial applications. An evaluation of the sustainability and of the feasibility of the new proposed materials for contaminants adsorption as a candidate for activated carbon substitution, is a fundamental topic for engineering applications and with potential massive economic returns.

In this context, it must be highlighted that an exact estimation of the market cost of a new material is highly difficult, because it depends on several factors, e.g., the market dimension and a cost quantification result reliable over a defined period of time.

For these reasons it is convenient to use other parameters, accounting energies and emissions related to the material synthesis, strongly dependent on material typology. In a very recent work (see Bontempi, 2017a) a new approach to quantify the sustainability of a raw material substitution, based on embodied energy (EE) and carbon footprint (CF) has been proposed.

The material production from ores and feedstock needs energy. It is defined the “embodied energy” and includes all energies (direct and indirect) consumed during the production of 1 kg of a specific material (Ashby, 2012). The CO_2_ footprint corresponds to the equivalent mass of greenhouse gases (kg CO_2_ equivalent), produced and released into the atmosphere because of the production of 1 kg of the material (Ashby, 2012). The EE accounts for the resources and the CF for the emissions, involved in a material production. It was recently shown (see Bontempi, 2017b) that EE and world materials production are correlated by an exponential curve, similar to the demand curve (in the economy context). Then the use of the material EE and CF (that are material dependent constant values) can be a suitable way to compare different materials, in terms of sustainability.

Data concerning EE and CF of materials considered in this work were obtained from CES Selector 2016 (Granta Design, Cambridge UK).

**Figure 9:**
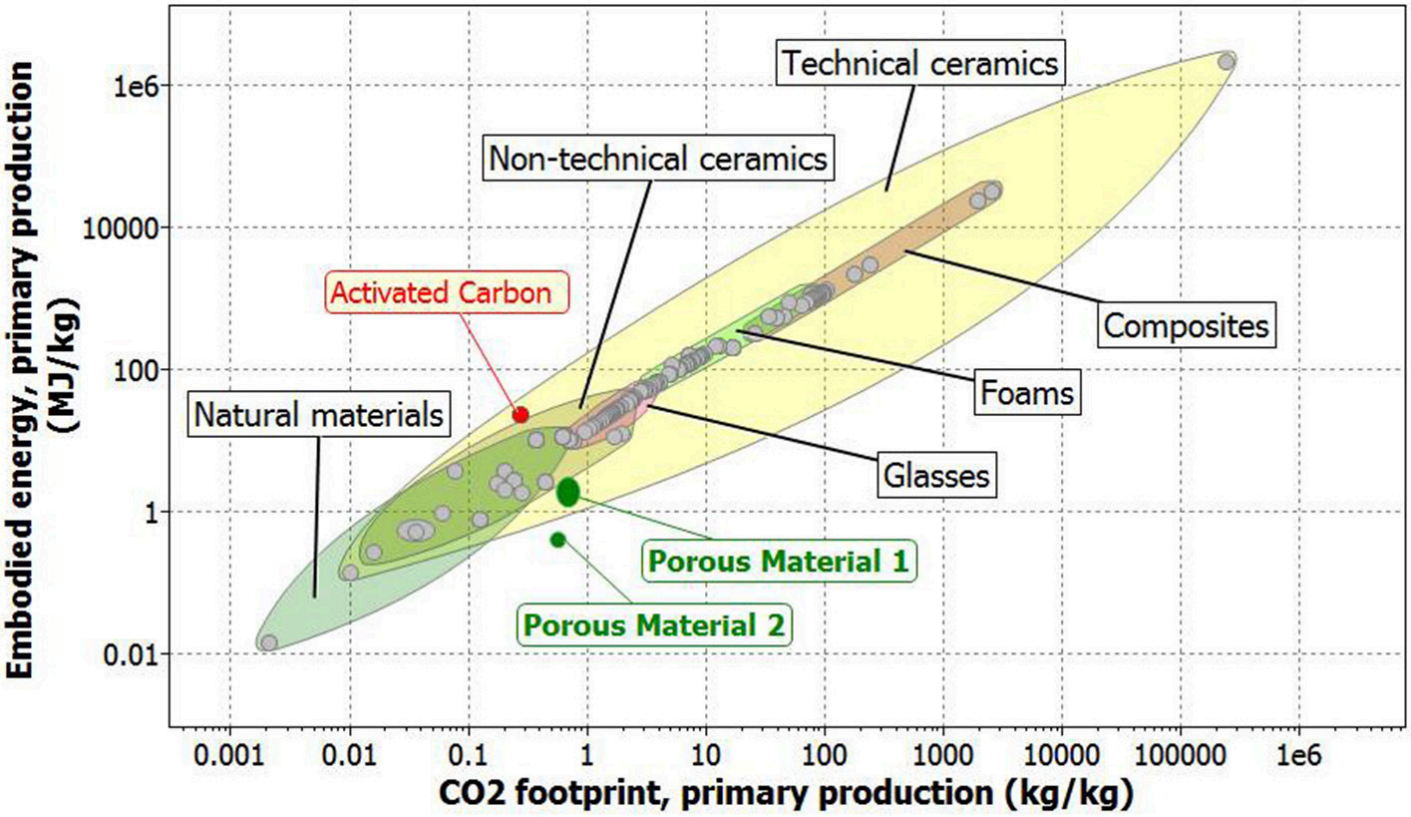
Embodied energy (EE) in respect to CO_2_ footprint (CF) of the synthesized materials, considering (porous material 1), and not considering (porous material 2) the thermal annealing. As a comparison, also ceramics and natural materials (natural resources).

Silica fume is often classified as a by-product material, which requires low amounts of energy for capture. For this reason, literature usually considers a zero energy allocation if their capture is attributed as a waste treatment process (Jamieson et al., 2015). A similar consideration is made for carbon footprint. However, sometimes silica fume is used in cement production, and thus a level of energy and emission associated to its supply, higher than zero, should be attributed to these by-products. For example, in some publications this value is set at 0.1 MJ/kg (Jamieson et al., 2015). In this case, it is chosen to recommend higher conservative maximum values to EE and CF (respectively 0.16 MJ/kg and 0.02 kg/kg), in accordance to tabulated CES values. As a result, the EE of the porous material here presented is comprised between 1.25 and 3.01 MJ/kg, and its CF values are between 0.55 and 0.85 kg/kg. This large variability is essentially due to the energy source used for thermal annealing of sample (ranging from electrical to petroleum origin) to obtain sodium bicarbonate decomposition. Moreover, it is also possible to use solar energy to obtain thermal treatment, by leaving the sample outdoor during sunny days as already demonstrated on different trials. This experiment was successfully carried out by leaving the sample outdoor, and by exposing it to the solar light for 30 min, in July 2017 (45°32′08″N−10°12′52″E). EE and CF of the obtained porous materials are reported in Figure 9. For the sake of comparison also EE and CF of ceramic materials, foams, and natural materials, are reported (CES Selector). Also activated carbon energetic and emissive parameters are reported. From data reported in Figure 9, it becomes evident that the new proposed material results more convenient in terms of emissions and energies required for synthesis, in comparison to activated carbon.

According to Bontempi (2017a), the SUB-RAW index was calculated. This made it possible to quantify the sustainability material with respect to natural resources. In effect, the SUB-RAW indices result 0.32 and 0.70, respectively considering and not considering a thermal treatment. This means that the new proposed porous material is more sustainable, in comparison to activated carbon. This is mainly due to the difference in EE of the two materials that corresponds about one order of magnitude. Finally, to evaluate the efficiency of the new proposed material in comparison to activated carbon, the porous material was reduced in powder form. Indeed, it is well-known that a material in powder form is more efficient in respect to the same materials, synthesized in bulk form (like the disks used in this paper), for the adsorption. The removal efficiency, calculated by Equation (1), resulted 54% and 99.5% for porous material (reduced in powder form) and activated carbon, respectively. This result is extremely important, because it highlights the good adsorption characteristics that can be obtained, by a new material, more sustainable in respect to natural resources (like activated carbon). In conclusion, considering the data reported in Figure 9 (concerning EE and CF of some materials used for adsorption) and the SUB-RAW index, it is possible to normalize these values, considering the materials adsorption properties. In this case the efficiency of the new proposed material results about 54% in respect to that of activated carbon. However considering a mass normalization, the SUB-RAW index results positive (0.05 and 0.43, respectively considering and not considering a thermal treatment for the synthesis of porous material), indicating an environmental and energetic advantage in the activated carbon substitution.

## Conclusions

The aim of this paper was the development of a new sustainable porous material obtained by an industrial by-product (silica fume) and abundant organic raw materials (sodium alginate). The extensive material characterization demonstrated that the material, which can resemble an organic-inorganic hybrid can be efficiently used to remove organic dyes like methylene blue, even at high concentration with efficiency up to 94%. By coating the material with a thin film of titania the material showed a good photodegradation performance.

A striking feature is that the material does not require a thermal treatment at high temperatures for stabilization, but it is indeed self-stabilizing. Promising ongoing tests indicate that the material store at ambient conditions becomes more stable over time and no degradation takes place. The capability of the new material to capture diesel exhaust PM is also shown. Then it may be also applied as an external coating of buildings to reduce the concentration of PM in urban areas.

Material sustainability was evaluated and successfully demonstrated considering embodied energy and CO_2_ footprint as key parameters. On the base of these two parameters, it is demonstrated that it is possible to synthesize a material for pollutants removal, more sustainable than activated carbon, which represents the gold standard.

Finally, the possibility to shape the material by 3D printing highlights its high versatility and opens new possibilities. For example, filters, components with specific dimensions and geometries or replacement parts can be easily designed and manufactured and integrated in the plant. Indeed, 3D printing allows to realize personalized designed components, that may be not available on the market, or that may have high production costs, and require time to be delivered.

## Author contributions

EB conceived of the presented idea and supervised the project; AZan, SF, EV, MM, and AZac carried out the experiments; TM and IV supervised the project and contributed to the final version of the manuscript; EB and LT wrote the manuscript, with support of AZan. All authors discussed the results and commented on the manuscript.

### Conflict of interest statement

The authors declare that the research was conducted in the absence of any commercial or financial relationships that could be construed as a potential conflict of interest.
